# Bleaching Sponge

**Published:** 1865

**Authors:** 


Bleaching Sponge.—I recollect seeing a few months since in your journal some processes for bleaching sponge, but I think in each case the sponge had to be immersed in an acid solution. I have found the following a better way, inasmuch as nothing except gaseous chloride comes in contact with the sponge. In the first place, I get a box and a basin; I then put some sponge on a flat surface, and invert the box over it; in the basin I put some "chloride of lime" and sulphuric acid, and place it under the inverted box. The chlorine becomes quickly disengaged, and in a few minutes imparts to the sponge a nice white appearance, apparently without the slightest injury to its texture. The operation had better be performed in the air, as it soon fills the room or house with a powerful smell of chlorine.—A. W. S., in the *Chemist and Druggist.*

